# Network Models for Cognitive Development and Intelligence

**DOI:** 10.3390/jintelligence5020016

**Published:** 2017-04-20

**Authors:** Han L. J. van der Maas, Kees-Jan Kan, Maarten Marsman, Claire E. Stevenson

**Affiliations:** 1 Psychological Methods, University of Amsterdam, Nieuwe Achtergracht 129B, 1018 WX Amsterdam, The Netherlands; M.Marsman@UvA.nl (M.M.); C.E.Stevenson@UvA.nl (C.E.S.); 2 Research Institute of Child Development and Education, University of Amsterdam, Nieuwe Achtergracht 127, 1018 WS Amsterdam, The Netherlands; K.J.Kan@UvA.nl

**Keywords:** intelligence, development of intelligence, cognitive development, network models, factor models, psychometrics, latent variable models

## Abstract

Cronbach’s (1957) famous division of scientific psychology into two disciplines is still apparent for the fields of cognition (general mechanisms) and intelligence (dimensionality of individual differences). The welcome integration of the two fields requires the construction of mechanistic models of cognition and cognitive development that explain key phenomena in individual differences research. In this paper, we argue that network modeling is a promising approach to integrate the processes of cognitive development and (developing) intelligence into one unified theory. Network models are defined mathematically, describe mechanisms on the level of the individual, and are able to explain positive correlations among intelligence subtest scores—the empirical basis for the well-known *g*-factor—as well as more complex factorial structures. Links between network modeling, factor modeling, and item response theory allow for a common metric, encompassing both discrete and continuous characteristics, for cognitive development and intelligence.

## 1. Introduction

In this paper, we introduce a new unified model of general intelligence based on network modeling. This model integrates different well-known explanations of the positive correlations between intelligence subtest scores and explains many key phenomena in intelligence research. The links between network models and psychometrics allow for novel developmental interpretations of well-known psychometric models and integrate both the measurement and understanding of cognitive development and intelligence. We argue that this link is instrumental in bridging Cronbach’s famous division of scientific psychology into two disciplines.

The study of cognitive performance is an excellent example illustrating the relevance of Cronbach’s famous division of scientific psychology into two disciplines, that of mechanisms and that of individual differences [1]. Cronbach noted that: “It is now commonplace for a student to get his PhD in experimental psychology without graduate training in test theory or developmental psychology, and the student of correlational branches can avoid experimental psychology only a little less completely” [1] (p. 672). This was true in 1957 and remains true 60 years later. Presenters at conferences on intelligence will rarely meet the visitors of conferences on cognition. Journals such as Intelligence, Structural Equation Modeling, Learning and Individual Differences, on the one hand, and journals such as Cognition, Learning, Memory & Cognition, Brain, and the Journal of Mathematical Psychology, on the other hand, represent very different approaches, but ultimately aim to understand the same thing: how our minds work.

The main reason for this division is that the two underlying questions, the mechanisms of cognition and the sources of individual differences in cognition, are very different questions that can largely be answered without reference to each other. As Cronbach puts it: “Just as individual variation is a source of embarrassment to the experimenter, so treatment variation attenuates the results of the correlator” [1] (p. 674). We can have a perfect explanation about how humans in general solve arithmetic problems without having a clue about the reasons why some people do better than others, and the other way around, knowing an important source of individual differences in, say, playing chess (e.g., amount of training) tells us little to nothing about the mechanism (i.e., how we play chess). 

Even worse, many mistakes in psychological science are due to incorrect generalizations across these two fields. General intelligence is a famous example. The fact that individual differences in intelligence subtest scores can be modelled with one general factor does not imply that there is one general intelligence module in the brain [2,3]. Another more subtle example is the mistake of using measures developed for experimental research (e.g., to detect differences between experimental conditions) as instruments to assess individual abilities—the latter case requires much higher reliability. This is, for example, a problem for the measurement of creativity [4]. 

Cronbach was rather optimistic about the unification of these two disciplines in psychology [1], but 60 years later the division is still apparent. Our take on this is that unification requires models of the process within individuals—the mechanism, i.e., how it works—augmented with assumptions about sources of individual differences. Ideally, we would have a cognitive model of, say, how people solve multiplication problems, with, for instance, the added assumption that working memory is the main source of individual differences in solving performance. 

With this idea in mind, we first review three models of general intelligence: the *g*-model, the sampling model, and the mutualism model. We argue that the mutualism model, a network model of cognitive capacities and abilities, although very primitive, has the necessary elements in place to provide a more unified approach to cognition and intelligence. We then propose a new unified network model for general intelligence that combines explanations based on sampling, mutualism, and central processes. This network model of how general intelligence operates within the individual can explain major phenomena in the field of intelligence—both within and between persons.

Second, we discuss the relationship between network models, such as the mutualism model, and modern psychometrics in order to demonstrate how a common metric can be constructed to measure both cognitive development and individual differences in intelligence. Networks are complex systems that sometimes exhibit phase transitions—reminiscent of stages in cognitive development in the current context—implying discrete latent variables, such as those in latent class analysis. In contrast, most current day psychometrics generally uses continuous latent variable models to measure individual differences. Common measurement is supported by mathematical links between various network model approaches and psychometric models, such as factor models and item response models. We describe the links between these two types of models and demonstrate how the same network can give rise to both continuous and discontinuous cognitive-intellectual development depending on network characteristics.

## 2. The Factor Model Dominance 

One of the most influential differential psychologists of all time is Charles Spearman, not because of the rank correlation coefficient that is named after him, but because of his pioneering work on factor analysis and its application to intelligence research. Spearman’s idea of a realistic general intelligence, or “*g*” that explains individual differences in cognitive abilities [5], still dominates intelligence research. Both in common sense and in research the basic idea of an underlying *g*-factor is tremendously popular. Moreover, the factor analytic way of thinking about individual differences, developed in intelligence research, is nowadays the prevailing approach in many areas of differential psychology. Psychologists routinely develop tests for some construct, apply structural equation modeling, and conclude that individual differences in observed scores are due to one or more underlying variables, the common factors in the structural equation model. The reasoning is basically that the positive correlations between observed variables are due to some underlying psychological (or biological) attribute, such as intelligence, depression, extraversion, or leadership. 

There are some evident advantages of this approach to differential psychology. First, latent variable modeling is statistically well developed. After Spearman introduced the one factor model [5], models have been developed for multiple latent variables (e.g., Cattell’s influential model of fluid and crystallized intelligence [6]), hierarchical structures, time-dependent relations, and nominal and ordinal data. These models can be estimated with different methods available in many software packages. Latent variable models allow for the assessment of model fit, model comparison, the assessment of measurement equivalence, tests of differential item functioning, and much more. 

Second, the latent variable approach is a very useful data reduction technique. We can easily collect tens of measurements on psychological constructs and from a practical point of view it is desirable to then summarize these into a limited set of variables, which is possible when all these measures correlate reasonably well (the so-called positive manifold and most important phenomenon in cognitive ability research). Bartholomew convincingly argues that the latent variable approach is superior to the simple summation of variables used to compute traditional IQ-scores [7]. 

Third, the latent variable approach is straightforward and practical. For a number of reasons, it is extremely difficult to develop highly valid and reliable instruments in psychology; it is much easier to create multiple suboptimal ones. Take self-reporting as an example. Everybody understands that self-reports on, say, personality are vulnerable to numerous validity threats. According to Borsboom and colleagues, a test can validly measure an attribute if and only if variation in the attribute causally produces variation in the measurement outcomes [8]. This is hardly the case when people report on their conscientiousness. Perhaps the tidiness with which they handle the paper and pencil questionnaire is a better measure than the actual answers given, but often we have nothing better. Intelligence measures are also suboptimal in many ways. For instance, training can seriously bias its measurement [9]. The benefit of the latent variable approach is that quantity can compensate quality. Given that a certain psychological construct actually exists (more about this below) and given many different suboptimal measurements, the factor model approach gives accurate estimates of the real values for the construct in question. Since creating questionnaires and simple tests of mediocre reliability and validity is relatively easy, and applying the factor model requires only a few clicks in SPSS, this approach can be applied by any psychology researcher with a little training in statistics. 

So, what is the disadvantage? The main problem with the latent variable approach is that it is often unclear how we should interpret the latent variable. What is *g*? Is it mental power, brain size, working memory, speed of information processing in the brain, or something else? For that matter, what does the extraversion factor represent? The question concerning the status of the latent variable is extremely tricky, and different responses have been given. 

**Figure 1:**
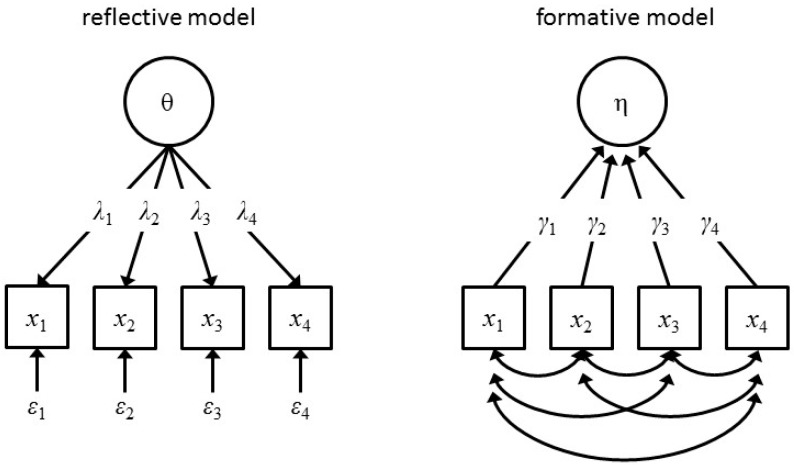
The reflective and formative latent variable model. In the reflective model (left) the latent variable (e.g., temperature) causes the manifest scores (e.g., thermometer values at different locations and times). In the formative model (right) the latent variable (e.g., economical index) summarizes the manifest scores (e.g., economic success of different companies).

One option is to discard the question. It is remarkable that in thousands of publications on factor models of general intelligence this basic question is largely ignored. In Jensen’s famous book on the *g*-factor, he never really answers this question (“a biologically based variable”) [10]. Some researchers take a practical point of view: the *g*-factor, whatever it is, predicts educational and job success [11]. Others take a statistical point of view and argue that they are not obliged to take a position about the ontological status of latent variables [12]. We do not think these positions are satisfying. Explaining all relations between observed variables by one unobserved unidentified common cause reminds us of another theoretical framework that has not been scientifically productive. A “non-religious” application of the latent variable model requires answers to two important questions. First, we have to decide whether we will interpret the latent variable in a formative or reflective way (see Figure 1). Second, if we choose the reflective model, we have to answer the question about the origin and causal nature of the latent variable.

The difference between the reflective and formative interpretation of the latent variable is of fundamental importance. In the reflective model the latent variable is the true cause of the scores on the observed variables. The classic example is the thermometer. Suppose we have a set of thermometers of mediocre quality. When we collect sets of measurements at different points in time or in different locations we can then subject the data to factor analysis. The resulting factor will represent temperature, a physical realistic variable that is the source of the differences in measurement values on each of the thermometers across the locations. In contrast, in the formative model the latent variable is just an index of some complex system. Economic indexes are excellent examples; they summarize an economic situation, but differences between different companies’ indices obviously do not cause the differences in their economic success—it is the other way around.

We believe that the reflective model requires hypotheses about the origin and causal effects of the latent variable. For the *g*-factor model of general intelligence, such hypotheses have been put forward, but none of them have been accepted [13].

The formative model should therefore also be considered. This model is less attractive, as it does not explain anything in itself; the correlations between indicators are just there without explanation. In the next section, we describe models of general intelligence that conform to the formative model, but are reflective in that they introduce (alternative) causal explanations. They were developed within the context of general intelligence, but are relevant to many areas where the factor model is routinely applied. 

## 3. Alternative Explanations for the Positive Manifold of Cognitive Abilities

### 3.1. The Sampling Model

Bartholomew, Deary, and Lawn (2009), and more recently Kovacs and Conway (2016), re-introduced the sampling theory of general intelligence originally proposed by Thorndike (1927) and Thomson (1916) [14,15,16,17]. In the sampling model, positive correlations between test scores are due to the fact that any two cognitive tests will always share some underlying basic processes. This overlap in shared basic processes will necessarily result in positive correlations between tests. In the sampling model, the positive manifold is essentially a measurement artifact. If we could create tests that only measure the intended attribute and no other attributes, the positive correlations between tests would disappear. 

Bartholomew and colleagues generalized Thompson’s (1951) model to account for multiple latent factors [14]. Kovacs and Conway proposed a new version of the sampling model that accounts for both domain-general executive processes and more domain-specific processes [15]. 

Sampling theory is largely ignored in the intelligence literature, but it is not easily dismissed. Strictly unidimensional tests appear impossible to develop [18], and sampling from the same underlying processes or capacities seems inevitable. In addition, sampling may also play a role in the relation between genes and intelligence [19,20]. Cannon and Keller’s watershed model describes how specific genes influence “upstream” narrowly defined endophenotypes, which in turn play a role in upstream cognitive abilities [20,21].

### 3.2. Network Models

Van der Maas and colleagues introduced a completely different explanation for the positive manifold [13]. They developed a mathematical mutualistic network model inspired by research in eco-system modeling, where the dynamics are best described by a network of reciprocal causal effects. The idea of the mutualism model of general intelligence is that such reciprocal causal effects also occur during development. We can think of the cognitive system as a large set of basic capacities or abilities, and growth in one aspect is partly autonomous and partly based on growth in other aspects. A good example is the positive influence of language on cognition, and vice versa (examples are syntactic bootstrapping [22], and semantic bootstrapping [23]). Other examples are the relations between cognition and metacognition [24], action and perception [25], and performance and motivation [26].

This idea can be formalized in many different ways. Van der Maas applied the mutualistic Lotka-Volterra model [27], a mathematical model for population dynamics [13]. The cognitive system is assumed to comprise of W processes (e.g., working memory, reasoning ability, vocabulary, etc.—indexed with *i*). Change or growth in a process (*x_i_*) is modeled as a function of autonomous growth, i.e., growth that is not dependent upon other processes, and the influence of other processes. The first term of Equation (1) models the autonomous growth of each sub-process, this is a typical *s*-shaped logistic growth equation where *a_i_* specifies the growth rate for a particular process and *K_i_* indicates the upper limit in the growth of that particular cognitive process. The second term of Equation (1) models the mutualistic interactions between the different processes. The strength of the interactions between processes is represented in the interaction matrix ***M***, which is assumed to be equal for all people. The second equation defines *K_i_* (the process-specific limiting capacity) as a linear function of genetic (*G_i_*) and environmental (*E_i_*) factors (see [13] for details). The full model is:

(1) $$\frac{dx_{i}}{dt}=a_{i}x_{i}\left(1-\frac{x_{i}}{K_{i}}\right)+a_{i}\overset{W}{\underset{\begin{array}{c} j=1\\ j\neq i \end{array}}{\sum }}\frac{M_{ij}x_{i}x_{j}}{K_{i}}\;for\;i=1..W$$

(2) $$K_{i}=c_{i}G_{i}+\left(1-c_{i}\right)E_{i}$$

Given some low initial states, *x*_0_, the ability within each process *x_i_* will grow over time until it reaches a state of equilibrium. Individual differences in *x_i_*, or composites of *x* (such as IQ or *g*), are due to individual differences in limiting capacities *K_i_*. In the proof and simulation of the positive manifold, the *K*’s are uncorrelated between the cognitive sub-processes, which means that there is no single source of individual differences. 

For those not used to these types of equations this model might appear complex, but it is overly simplistic. To name a few aspects, ***M*** most likely differs between subjects because not all growth processes will start at the same time, and the linear model for the genetic and environmental impact ignores statistical interactions between the two. However, there are two major advantages. The first advantage is that this model can easily be investigated using both simulations and formal proofs. Van der Maas and colleagues demonstrated (analytically) that the equilibria of Equation (1) only depend on ***M*** and ***K***, and that a positive covariance matrix always characterizes these stable states [13]. For example, a mutualism model with equal interactions *M_ij_ = c* and a one factor model with equal factor loadings imply exactly the same covariance matrix.

The second major advantage is that the mutualism model explains the positive manifold of correlations between cognitive ability tests—a major phenomenon in intelligence research. In fact, van der Maas and colleagues showed that a number of phenomena in the study of general intelligence can be explained by this basic model [13]. For example, the hierarchical factor structure of intelligence, the low predictability of intelligence from early childhood performance, the integration/differentiation effect, and the increase in heritability of *g* can all be explained by the mutualism model. Also, the mutualism model only requires sparse and weak interactions (some even being negative) between cognitive processes to produce positive manifold data [13,28]. This is important, since transfer in cognitive development is often weak and limited to related abilities [29]. 

### 3.3. g, Sampling, and/or Mutualism

Above, we discussed three types of models that offer different explanations for the positive manifold of general intelligence. Evaluating and choosing between these models is not a simple task. It could be the case that all three explanations play a role in general intelligence. 

The main criticism of the *g*-factor model is that a century of research has not brought any consensus on the origin of *g*. This means that we do not know how *g* results in intelligence test scores, how *g* operates in the brain, or how *g* develops. In terms of Cronbach’s division of psychology disciplines, *g*-theory is an extreme case. It does a good job of explaining individual differences, but it provides no clues whatsoever concerning the architecture and development of the cognitive system. 

Sampling theory has been criticized by Jensen and Eysenck [10,30]. They claim that although some complex mental tests highly load on the *g* factor, as predicted by sampling theory, other very narrowly defined tests also display high *g* loadings. Second, some seemingly unrelated tests, such as visual and memory scan tasks, are sometimes highly correlated, while tests that are clearly related, such as forward and backward digit span, are often only modestly correlated. Third, while sampling theory can predict general impairments in cognitive functioning, brain damage sometimes leads to very specific impairments. However, it is a little uncertain whether the empirical claims underlying these criticisms are robust. Moreover, Kovacs and Conway argue that the sampling model is able to predict specific patterns of intercorrelations between different subtests of intelligence [15].

The mutualism model of intelligence [13] has also been criticized [30,31]. According to Gignac, the *g*-factor is stable from 3 years on, which he sees as an indirect rejection of the mutualism model because the positive manifold would be expected to develop throughout childhood and therefore show lower correlations between cognitive abilities at such a young age [31]. Gignac claims that it would be very unlikely that the pattern of mutually beneficial interactions between cognitive abilities across individuals arise precisely in a manner that their latent variable inter-associations can be accounted for by a single latent variable [32]. Gignac shows, using several datasets, that such positive residual correlations do not occur. Van der Maas and Kan provided a rebuttal to these criticisms, showing that Gignac’s main premise is incorrect [28].

A second criticism of the mutualism model comes from Nisbett and colleagues, who claim that it (and the sampling model) fail to distinguish between genetic and environmental effects [33]. This is only partly true. As shown in Equation (2), genetic and environmental influences are accounted for in ***K***, the person-specific limiting capacity determined by genetics (***G***) and the environment (***E***); this naturally explains the increased influence of heredity with age on intelligence, but the authors acknowledge that the role of an external environment is missing [13]. However, this can be accounted for with Dickens’ multiplier model, which is very similar to the mutualism model [34,35]. In this model, some of the reciprocal causal effects are routed through the environment. Strong performance in one domain (Dickens uses basketball as an example) leads to environmental support (in the form of better training), which in turn leads to even better performance. These multiplier effects can also occur across domains. In this way, the mutualism/Dickens model incorporates gene by environment interactions and possibly explains the so-called Flynn and Jensen effects [36]. 

We started this section with the idea that all three explanations may play a role in general intelligence. First, although sampling theory is largely ignored in the intelligence literature, it seems to be at least partly correct because making truly unidimensional tests is likely impossible [18] and sampling from the same underlying processes between tests seems inevitable. Second, it seems implausible that there would be no mutualistic effects of growth between different cognitive abilities. Van der Maas and colleagues have already shown that very small and even partly negative interactions can lead to the typical positive manifold data that gives rise to prominent factor-analytic models found in the intelligence literature [13]. Third, even if we conceptualize general intelligence as a network of cognitive abilities, some of these abilities will have a more prominent role. In real networks, nodes also differ in centrality, meaning that some nodes have more influence on neighboring nodes than others. It is very plausible that, for instance, working memory has a more central role in the network of cognitive entities that make up general intelligence. In this case, the correlation between a working memory factor and the *g*-factor could be close to one. 

**Figure 2:**
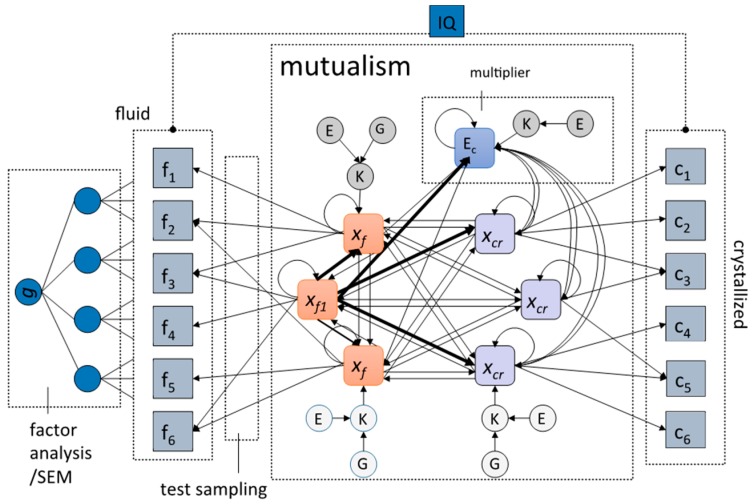
The unified model of general intelligence allowing for test sampling, reciprocal effects (both mutualistic and multiplier), and central cognitive abilities (such as working memory, *x_f_*_1_). The *x_f_* and *x_c_* nodes represent separate cognitive abilities in the intelligence network. The *c_i_* and *f_i_* represent test results of crystalized and fluid cognitive abilities, respectively, the sum of which is IQ. The *g*-factor can be extracted using factor analysis on *f* (and *c*) tests. See Equations (1) and (2) for more details on the internal workings.

**Figure 3:**
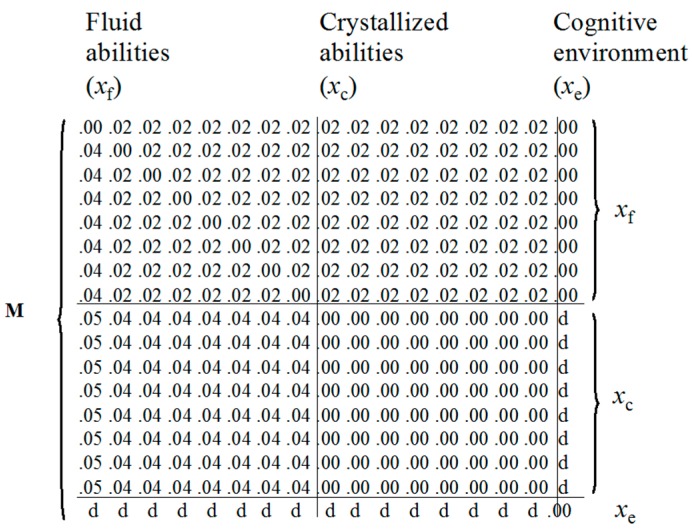
*M* matrix for the model in Figure 2.

Van der Maas and colleagues combined these insights and proposed the formal unified model of general intelligence that is depicted in Figure 2 [37]. Mutualism forms the core of this model, and the addition of a node representing the environment incorporates Dickens multiplier idea, which also incorporates the training of abilities (see [34]). We account for Cattell’s investment theory [6,38] by separating fluid and crystalized abilities. We also included the idea that some fluid processes are more central (*x_f_*_1_) than others, reflected by stronger connections to other nodes. Such central processes will correlate very strongly with the *g*-factor extracted from a factor analysis of the separate cognitive ability test scores. All of these explanations can be included into a unified network model (Figure 2) with the specification of ***M***, depicted in Figure 3. 

Kan demonstrated through simulations that this model can also explain the Jensen effect, which, remarkably, is based on higher heritabilities and higher *g*-loadings for crystallized tests such as vocabulary and arithmetic [37,39]. Sampling is included as an explanation at the measurement level of the model. Here, we assume that all tests tap from different abilities at the same time, forming another source of covariation between test scores. Sampling is the most obvious explanation, for example, for the high *g*-loadings of tests like the Raven Progressive Matrices, which require multiple abilities to solve. 

One attractive property of this model is that it is a developmental model of the individual, but also explains effects across individuals, such as the positive manifold. It includes several explanations of how general intelligence emerges and explains phenomena related to individual differences. With these important aspects, it makes large strides in integrating the two disciplines distinguished by Cronbach [1]. 

In the remainder of this paper we discuss several implications of using networks as a basic model for general intelligence.

## 4. Network Psychometrics

Complex systems are studied in fields such as mathematics, physics, chemistry, biology, and economics, and the human mind is probably the most complex system studied in all of science. Networks (or graphs) are a common framework for studying complex systems. Network science is a very productive area of research and has recently become popular in psychology, with applications mainly in clinical psychology and in social psychology [40,41]. The general hypothesis is that when observed behaviors, such as cognitive abilities, psychopathological symptoms, or attitudes, occur together it is not due to unobserved common causes, such as general intelligence, psychopathological disorders, attitudes, or personality traits, but rather due to behavior that emerges from a network of interacting psychological and/or biological factors. 

This approach has led to a number of new insights concerning, for instance, the co-morbidity of clinical symptoms [42], early warnings for clinical disorders [43,44], and the stability of attitudes [41,45]. These insights are based on network methods using new statistical analysis and visualization techniques [46,47,48]. The most popular models that are used for cross-sectional data are the Gaussian Graphical Model (GGM) for continuous data [49], the Ising model for binary data [50], and their combinations [46,51]. Changes in networks over time can be estimated using vector-auto-regression (VAR) [52]. Furthermore, many other methods are also being developed ([53,54] for an overview of what is now called network psychometrics, see [55]). 

**Figure 4:**
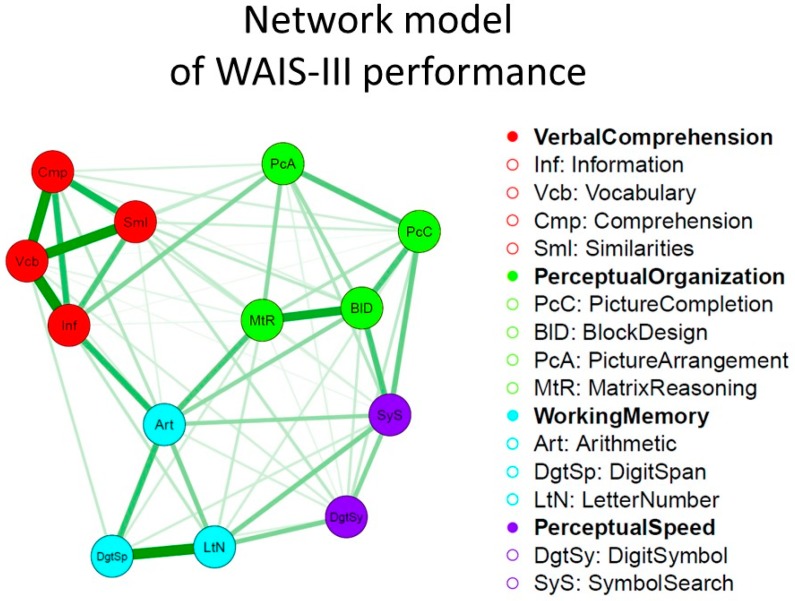
Basic network visualization of the WAIS-III intercorrelations [56], using qgraph [48].

**Figure 5:**
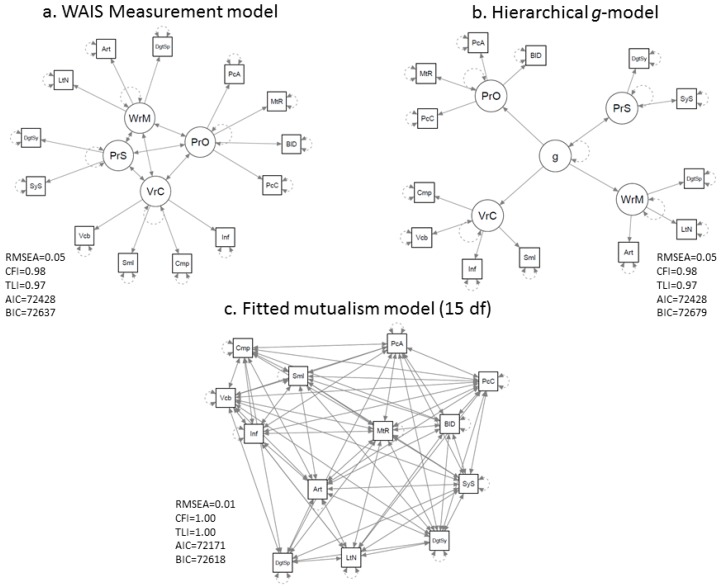
Depictions of models of the WAIS-III dataset [56] that were compared to the mutualism network model of intelligence: the WAIS-III measurement model (**a**) and the hierarchical *g*-factor model (**b**). The constrained mutualism network model (**c**) fitted the data best. The AIC and BIC for the saturated model were 72181 and 72709, respectively.

A simple example of a network visualization of intelligence data [56] is presented in Figure 4. It depicts a Gaussian graphical model of the mutualism network model of intelligence of the WAIS-III dataset (computed in qgraph [48] with the graphical lasso [57] and a tuning parameter chosen using the Extended BIC [58]). This model can be compared to traditional factor models by fitting a symmetrical mutualism model in which the nonsignificant mutualism weights are dropped from the model (constrained to 0). We considered various competing factor models. In Figure 5 we show the WAIS-III theoretical measurement model, a typical *g* theoretical second order factor model in which the positive correlation between the factors in the initial measurement model are explained by *g*, and constrained mutualism model, with the best fit for the latter model.

Networks exist in a huge variety. The Ising network model is particularly interesting for dichotomous data, which factor theories of intelligence are generally based upon.^1^ This model was introduced by Lenz to explain the phenomenon of magnetism in statistical physics [60,61], and consists of binary nodes that are connected in a network, where nodes that have a positive connection tend to be in the same state. Psychological variables may behave in the same way. For example, interactions between symptoms of depression can cause synchronized effects in the system as a whole (e.g., depression as a disorder). Also, knowledge facts, cognitive strategies, and other elements of a cognitive ability may be connected in a similar way. This implies that a latent trait, such as depression or ability, can be seen as a cluster of connected nodes [62]. 

Several papers describe the relationship between the Ising model and other statistical models, such as the loglinear model, logistic regression, collider models, and item response theory models [63,64,65,66], a relation that is based on the work of Kac [67]. In particular, these papers demonstrate the statistical equivalence between the Ising model and multidimensional item response theory (MIRT) models. A similar link exists between the mutualism model and the factor model (see Appendix of [13]). This equivalence has many consequences. It allows for novel interpretations of long-standing psychometric models, new estimation techniques, and a new conceptualization of measurement in psychology. This is very interesting in the context of this paper, as the link between the Ising model and MIRT establishes a link between models of processes and mechanisms (in the form of networks) and the statistical methods to analyze individual differences (psychometrics).

### Complex Behavior in Networks

The dynamics of the mutualism model as applied here are rather simple. Each of the cognitive components of the network show a typical s-shaped logistic growth pattern. The Ising model, under certain conditions, also behaves in an unspectacular manner. These cases will give rise to standard latent variable models, such as the “*g*”-factor, with continuous latent variables (e.g., cognitive ability test scores). This is already a very important insight. The standard justification for using (continuous) latent variable models, such as the factor model and item response theory (IRT), in differential psychology is essentially a practical one, for example, the logistic equation in IRT is justified in terms of mathematical elegance and practical use. The formal derivation of such models from network models provides an interpretation at the individual level, which is an important finding. 

Remarkably, the link between network modeling and psychometrics extends to categorical latent variables. Categorical latent variables, consisting of latent classes or mixture components, are especially relevant when we want to model individual differences in terms of classifications such as in developmental and clinical psychology (e.g., type of learning disability or personality disorder). The most important point to make is that with network modelling we can understand why the same psychological processes and systems sometimes behave according to continuous latent variable models (e.g., gains in an IQ-score) and at other times behave according to discrete latent variable models (e.g., progression through cognitive stages). 

The crucial point is that the Ising model and many other network models also exhibit phase transitions between alternative stable states, such as the transition from primary reasoning to understanding permission rules in the reasoning domain of Demetriou’s theory of cognitive development [68]. Network models are typical nonlinear dynamic systems characterized by complex behavior with alternative states of equilibrium. The occurrence of alternative stable states is especially relevant when it comes to development [69]. It is remarkable that Piagetian concepts, such as disequilibrium, stages, transitions, and reorganization, are all terms that have well defined meanings in complex systems theory. 

**Figure 6:**
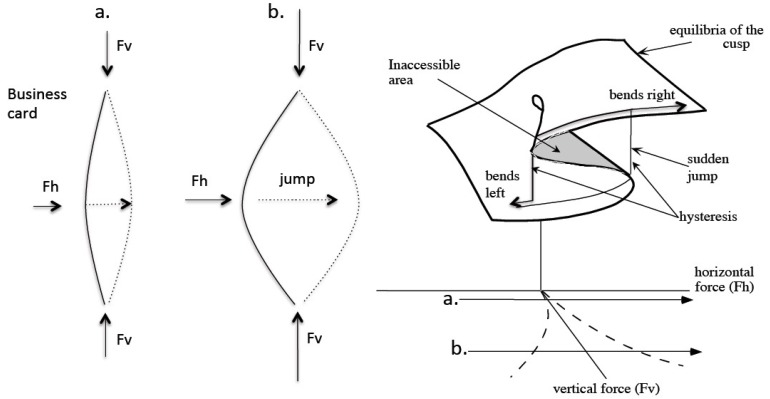
The cusp catastrophe that underlies stage transitions in cognitive development illustrated by the dynamics of a simple business card. Vertical pressure (*Fv*) on the card is the splitting variable (e.g., test conditions) and horizontal pressure (*Fh*) is the normal variable (e.g., instruction). With low vertical pressure (path **a**), switches in the middle point of the card are continuous, with high vertical pressure (path **b**) they are sudden, exhibiting hysteresis, bimodality, divergence, and other typical characteristics of phase transitions.

Catastrophe theory can help to investigate Piagetian phase transitions (e.g., [70,71]). Catastrophe theory (nowadays referred to as bifurcation theory), particularly cusp catastrophe, classifies phase transitions in complex systems—such as those in physics, chemistry, and biology. The cusp catastrophe describes sudden transitions in a behavioral variable caused by smooth and slow changes in two control variables, the normal and splitting variable. Figure 6 illustrates the cusp catastrophe using the analogy of sudden switches in a simple business card.

Cramer and colleagues discuss the relationship between a psychological network model of depression and the cusp catastrophe [72]. Depression is modeled as a symptom network. Vulnerable symptom networks are those with strong connections between the symptoms. Stressors, *S*, may push the network to a depressed state in which most symptoms are “active”—resulting in depression. The probability, *p_i_*, of symptom *X_i_* being active at time *t* is modeled by a logistic function:

(3) $$\log\left(\frac{p_{i}^{t}}{1-p_{i}^{t}}\right)=-b_{i}+\sum_{j=1}^{J}cM_{ij}X_{j}^{t-1}+S_{i}^{t}$$

***M*** is again a weight matrix of connections, *c* is general connectivity constant, and *b_i_* is the threshold of a symptom. A higher threshold means that a higher stressor (*S_i_*) is needed to activate the symptom. 

Cramer and colleagues not only show that stronger connections (higher *c*) increase the probability of a depressed state, they also demonstrate that stronger connections operate as a splitting variable [72]. That is, for low *c*, smooth changes in stressors lead to a continuous change in the sum of the symptoms (a typical way to measure depression). When *c* is high, this change is sudden; the network exhibits alternative stable states (i.e., depressed or not) and hysteresis. 

This is just one example of a phase transition in a psychological network. Phase transitions, such as those from a non-depressed to depressed state, can be explained using the Ising model. It was originally used in the study of ferromagnetism (i.e., how materials such as iron become magnets) to explain phase transitions (e.g., positive to negative polarity) in a field of particles. The state of each particle depends on that of the other particles. At a low temperature, all particles tend to synchronize leading to magnetization, whereas, at higher temperatures particles behave more randomly. At low temperatures, sudden transitions in magnetization may occur (e.g., from positive to negative polarity). The dynamics of the Ising model can be understood using the concept of energy. It seeks states with the lowest energy. Unaligned particles increase the energy of the system. This is summarized using the Hamiltonian function:

(4) $$H\left(X\right)=\;-\underset{i}{\sum }\tau_{i}x_{i}-\underset{\langle i,j\rangle }{\sum }M_{ij}x_{i}x_{j}$$

which, in the Gibbs distribution [27], implies the probability of ***X*** (e.g., a particle) being in some state ***x*** (e.g., positive), as shown in Equation (5):

(5) $$P\left(X=x\right)=\frac{\exp\left(-\beta H\left(x\right)\right)}{Z}$$

Here, ***τ*** are thresholds (analogous to the thresholds *b* in Equation (3)) possibly influenced by an external field, β is the inverse temperature parameter and related to the general connectivity parameter in Equation (3), and *Z* is the integration constant. 

The Ising model is equivalent to a special case of the multivariate 2-parameter logistic IRT, which has been demonstrated in several papers [54,63,64,65,66]. Here, the temperature relates to the item discrimination parameters in the 2-parameter logistic IRT model, which is conceptually similar to the item-total correlation—the association between the chance of solving an item correctly and that of solving the rest of the test correctly. High temperature or low discrimination implies more random response patterns. Low temperature, thus high discrimination, results in response patterns in which all items are answered either correctly or incorrectly. In the latter case, the latent score distribution diverges from a Gaussian distribution and becomes bimodal. Such a bimodal latent score distribution is required to model individual differences in terms of types or latent classes [73]. Thus, the same Ising model may imply a continuous or discrete latent trait model depending on the temperature parameter. We saw the same phenomena in the model of Cramer and colleagues, where behavioral change is discrete or continuous depending on the connectivity parameter [72]. It is an important insight that complex systems can continuously change between a state, where change is discrete to a state where change is smooth and continuous. 

This implies that individual differences also may appear discrete (in the form of latent classes) or gradual (in the form of latent traits) depending on parameters that may change over time. A beautiful example is found in the study of attitudes [41], where the “inverse temperature” can be equated with involvement or importance of the attitude for the person or in the society. In the case of low involvement, distributions of attitude scores are normal; in the case of high involvement, they are bimodal [45].

In the case of cognitive development, we predicted a similar role for optimal test conditions [71]. In suboptimal conditions, we expect much more gradual developmental curves and quantitative differences between people. In optimal test conditions, for example, when a child is assessed individually by a psychologist, we expect sudden changes and more qualitative individual differences (for a recent example see [74]). This, however, may not explain why some cognitive abilities often exhibit discontinuous change (classical Piagetian tasks are a prime example), whereas other cognitive abilities seem to develop more gradually (e.g., arithmetic). The distinction between two conceptually different IRT models, the Q-diffusion and D-diffusion model, proposed in [75], is highly relevant here:

(6) $$p\left(+\right)=\frac{e^{\frac{a^{p}v^{p}}{a^{i}v^{i}}}}{1+e^{\frac{a^{p}v^{p}}{a^{i}v^{i}}}}$$

(7) $$p\left(+\right)=\frac{e^{\frac{a^{p}}{a^{i}}\left(v^{p}-v^{i}\right)}}{1+e^{\frac{a^{p}}{a^{i}}\left(v^{p}-v^{i}\right)}}$$

The probability correct, *p(+)*, depends on two parameters defined in the drift diffusion model for decision making [76]. These are the drift rate, or accumulation rate of evidence, *v*, and the boundary separation *a*. The indexes *p* and *i* refer to persons and items, respectively. We refer to [75] for further explanation.

These IRT models, derived from the continuous drift diffusion model for decision making [76], differ with respect to range of the scale. The Q-diffusion model is mainly used for ability and only contains positive values for the person (and item) parameters, the lowest value being zero. In this model, response probabilities below chance level are excluded. The idea is that there is no such thing as negative ability (or difficulty). In the D-diffusion model, positive and negative person (and item) drift parameters are allowed and response probabilities range from 0 to 1. This model is primarily used for the modeling of affect and attitudes. In the Q-diffusion model, the zero point represents the absence of ability, whereas in the D-diffusion model, the zero point represents neutral affect or attitude. 

In [75], one exception is discussed. In some cognitive tasks, systematic below chance response probabilities are expected. In such tasks, misconceptions usually play a role. Based on a misconception, low ability subjects do not answer at chance level but systematically give wrong answers. Most Piagetian tasks share this property. For instance, non-conservers systematically choose the wrong option in a two-choice task based on the misconception that the glass with the highest water level contains the most liquid. In this case, the D-diffusion model is more appropriate than the Q-diffusion model that is the standard for most abilities. The D-diffusion model is actually very similar to the standard 2-parameter logistic IRT model, and thus the Ising model discussed above. Thus, we have a consistent theory about performance on typical Piagetian tasks. We assume the Ising network model (or some continuous variant) as a conceptual model. The child’s ability is based on a network of related (mis)conceptions. The state of this network can flip from incorrect to correct. Whether this transition is sudden or gradual depends on the temperature (strength of connections), which may be manipulated by testing conditions [71]. Psychometrically, this model implies a 2-parameter logistic IRT (D-diffusion) model, with either unimodal or bimodal distributed person parameters, depending on the discrimination parameters that directly relate to connection strength in the network model. However, for standard abilities (say arithmetic) the story is incomplete. We currently have no network that is mathematically equivalent to the Q-diffusion model for standard cognitive abilities. However, it is clear that Piagetian abilities are very different from most cognitive abilities, for which we do not expect discontinuous development. 

## 5. Discussion

In this paper, we proposed a new unified network model of general intelligence that incorporates four basic explanations: mutualism between basic cognitive processes during development, multiplier effects through the environment, sampling in manifest test scores, and centrality of key processes such as working memory. This unified model explains many phenomena in intelligence research. First, a number of phenomena were already incorporated in the original mutualism model. For example, well-established genetic effects such as the increase of heritability with age and the hierarchical nature of statistical *g*-factor models. Second, by incorporating the mechanism of sampling, it explains why complex tests, such a matrix reasoning tests, strongly load on the *g*-factor. Third, by allowing for some cognitive processes to be more central nodes, with working memory as a typical example, the high *g*-loading of such tests can be explained. Fourth and finally, the multiplier effect proposed by Dickens is also included. This way we can explain various genetic by environment correlation effects, such as the Jensen and Flynn effect. It is a novel idea to combine all these explanations into one model. It is very important that this model is formally specified, it is not a verbal theory (it is a “feory” not a “veory”). That means that its assumptions are completely clear, its predictions can be verified analytically and/or numerically, and unexpected and unrealistic side-effects can be identified. 

Network modeling is quickly becoming popular in psychology. Since 2006, when the original mutualism model of general intelligence was introduced, the network approach to psychological systems has developed conceptually and technically. Conceptually, networks are an attractive alternative for the dominant latent variable view on individual differences. With networks, it is not necessary to presume a common—but unidentified—cause of covariances between manifest variables. Technically, the network approach in psychology has been enriched with a number of data analysis tools. We did not give any examples in this paper, but shortly summarized the available techniques. We focused on the implications for the measurement of both individual differences in intelligence and the measurement of individual cognitive development. We explained the formal links between different types of networks and psychometric techniques, such as factor modeling and item response theory (IRT). As the network model is, in essence, a model of the individual, this link is of utmost importance. It provides a bridge between the two disciplines Cronbach described, from the world of how things work to the world of how things differ.

In the last part of this paper we have gone one step further. Latent variable models differ with respect to measurement scale: either continuous or discrete latent variables. In the first case, we usually use factor models (for continuous manifest variables) and IRT (for discrete manifest variables), which are commonly used to examine individual differences in intelligence. The equivalent techniques for discrete latent variables are, generally speaking, finite mixture models and latent class analysis. Discrete latent variables are used for stage-like change and typological individual differences, such as in cognitive development. 

We have shown that the same network can give rise to continuous and discrete behavior, depending on certain network parameters (that can vary continuously). The famous Ising network model, developed for magnetization, is formally equivalent to a multivariate 2-parameter logistic IRT model. Depending on the temperature, which is related to the strength of interaction between the nodes of the network, the resulting distribution of the latent trait is either unimodal or bimodal. Hence, the network approach also bridges the worlds of categorical and continuous latent variable models of individual differences. 

However, the unified network model is overly simplistic in some respects. We see many routes to more realistic and relevant network models. For example, in the present unified network model all nodes are there from the beginning, whereas cognitive development should also concern the actual growth of the network, where new nodes appear as new knowledge and abilities that are accumulated over time. 

Still, such a developing network is not the final word. In the world of cognitive modeling the (domain specific) models actually explain how problems posed in intelligence tests are solved. These models (connectionist, rule based, or hybrid) form the other end of Cronbach’s division of approaches to psychology. The network approach proposed in this paper concerns the collective operation of these domain specific mechanisms and, as such, helps to bridge the two worlds of intelligence and cognition.

## Footnotes

1 Though factor analysis on sum scores of subtests is most common, it is not a necessity. An alternative is item factor analysis [59], for instance. The positive manifold and “statistical” *g* can be investigated with both continuous and dichotomous scores. We focus here on the Ising model since it is very simple and the formal relation with a psychometric model has been established.
